# Ultra‐Sensitive, Deformable, and Transparent Triboelectric Tactile Sensor Based on Micro‐Pyramid Patterned Ionic Hydrogel for Interactive Human–Machine Interfaces

**DOI:** 10.1002/advs.202104168

**Published:** 2022-01-31

**Authors:** Kai Tao, Zhensheng Chen, Jiahao Yu, Haozhe Zeng, Jin Wu, Zixuan Wu, Qingyan Jia, Peng Li, Yongqing Fu, Honglong Chang, Weizheng Yuan

**Affiliations:** ^1^ Ministry of Education Key Laboratory of Micro and Nano Systems for Aerospace Northwestern Polytechnical University Xi'an 710072 P. R. China; ^2^ State Key Laboratory of Optoelectronic Materials and Technologies and the Guangdong Province Key Laboratory of Display Material and Technology School of Electronics and Information Technology Sun Yat‐sen University Guangzhou 510275 P. R. China; ^3^ Frontiers Science Center for Flexible Electronics (FSCFE) Xi'an Institute of Flexible Electronics (IFE) and Xi'an Institute of Biomedical Materials and Engineering (IBME) Northwestern Polytechnical University Xi'an 710072 P. R. China; ^4^ Faculty of Engineering and Environment Northumbria University Newcastle upon Tyne NE1 8ST UK

**Keywords:** flexible electronics, human–machine interface, micro‐pyramid‐patterned hydrogel, self‐powered hydrogel sensor, triboelectric tactile sensor

## Abstract

Rapid advances in wearable electronics and mechno‐sensational human–machine interfaces impose great challenges in developing flexible and deformable tactile sensors with high efficiency, ultra‐sensitivity, environment‐tolerance, and self‐sustainability. Herein, a tactile hydrogel sensor (THS) based on micro‐pyramid‐patterned double‐network (DN) ionic organohydrogels to detect subtle pressure changes by measuring the variations of triboelectric output signal without an external power supply is reported. By the first time of pyramidal‐patterned hydrogel fabrication method and laminated polydimethylsiloxane (PDMS) encapsulation process, the self‐powered THS shows the advantages of remarkable flexibility, good transparency (≈85%), and excellent sensing performance, including extraordinary sensitivity (45.97 mV Pa^−1^), fast response (≈20 ms), very low limit of detection (50 Pa) as well as good stability (36 000 cycles). Moreover, with the LiBr immersion treatment method, the THS possesses excellent long‐term hyper anti‐freezing and anti‐dehydrating properties, broad environmental tolerance (−20 to 60 °C), and instantaneous peak power density of 20 µW cm^−2^, providing reliable contact outputs with different materials and detecting very slight human motions. By integrating the signal acquisition/process circuit, the THS with excellent self‐power sensing ability is utilized as a switching button to control electric appliances and robotic hands by simulating human finger gestures, offering its great potentials for wearable and multi‐functional electronic applications.

## Graphical abstract

Triboelectric hydrogel tactile sensor is constructed based on micro‐pyramid‐patterned DN ionic organohydrogels with two working principles. This self‐powered tactile sensor with wide environmental tolerance and excellent sensing performance is obtained with a subtle immersion treatment and a micro‐pyramid‐patterned method. Combining with a signal acquisition/process circuit, wearable electronics and human–machine interface applications are demonstrated.

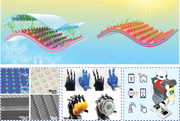

## 1 Introduction

Wearable and functional electronics, especially those of ultrasensitive, transparent, flexible, and stretchable devices, are urgently demanded by human beings.^[^ ^1^, ^2^, ^3^, ^4^, ^5^, ^6^, ^7^ ^]^ Various wearable devices based on mechanical sensors, flexible electronic skins, and artificial intelligence have been designed and introduced into our lives,^[^ ^8^, ^9^, ^10^, ^11^, ^12^, ^13^ ^]^ promoting the rapid evolution of human sciences and technologies. Combined with the integrated circuits and human–machine interface system, the transparent, deformable, and microscale mechanical sensors demonstrate their promising prospects for the future intelligent sensor system,^[^ ^14^, ^15^, ^16^, ^17^ ^]^ robot manipulation,^[^ ^18^, ^19^, ^20^, ^21^ ^]^ and digital twin applications.^[^ ^22^ ^]^ Different types of flexible electrodes or devices have been widely investigated for their merits of easy integration with wearable devices,^[^ ^23^, ^24^ ^]^ outstanding biocompatibility,^[^ ^25^ ^]^ and mechanical characteristics.^[^ ^26^ ^]^ However, there are still many issues such as power supply limitation, environmental pollution, poor flexibility, and difficult maintainability, which severely restrict their large‐scale applications in the internet of things (IoT).^[^ ^27^, ^28^, ^29^ ^]^ Hence, it is proposed that harvesting energy from external environments or human movements could become feasible to power such wearable sensors or devices.^[^ ^30^, ^31^, ^32^, ^33^, ^34^ ^]^

Noticeably, triboelectric nanogenerator (TENG) has attracted enormous attention for providing considerable power sources among various energy harvesters, with merits such as simple structures, low cost, vast materials to choose from, and efficient energy conversion rates.^[^ ^35^, ^36^, ^37^ ^]^ Principles of the TENG coupled with triboelectrification and electrostatic induction were first proposed by Prof. Zhonglin Wang's group in 2012.^[^ ^38^ ^]^ When two materials are contacted, electrons can migrate from one material to the other due to their different electron attraction abilities. With the continuous contact/separation of two materials induced by external stimulations, redistribution of electrons happens through using an external circuit, thus generating the alternating current and charging the small electronics. Various kinds of TENGs have been proposed to deal with the power demands of wearable and multi‐functional electronics in different aspects, including 3D printed stretchable harvesters,^[^ ^30^ ^]^ hierarchically structured generators,^[^ ^39^, ^40^ ^]^ surface‐modified TENGs,^[^ ^41^, ^42^, ^43^, ^44^ ^]^ self‐powered sensors with advanced material synthesis,^[^ ^45^, ^46^ ^]^ intelligent TENGs integrated with novel digital twin acquisition technology,^[^ ^6^, ^47^ ^]^ and optimization of power management circuits for TENGs.^[^ ^48^ ^]^

However, there are still significant challenges for these approaches:

- For example, the common electrodes used in TENGs are metals such as Cu, Al, or Au to ensure good conductivity, making the whole device rigid and opaque.^[^ ^23^, ^49^, ^50^ ^]^ Some relatively rigid while still flexible plastics (such as polyethylene terephthalate, polytetrafluoroethylene) are commonly utilized as flexible substrate.^[^ ^4^ ^]^ However, they often encounter difficulties for wearable and stretchable applications and bring discomfort and poor aesthetics to the human body.
- Most of the previously proposed flexible TENGs have poor environment‐tolerance problems, such as severe freeze or dehydration under some extreme temperatures due to the single function materials (such as nanoparticles or liquid metal).^[^ ^30^, ^46^, ^51^ ^]^ Moreover, good transparency, high deformability, and stretchability of the devices could not be easily realized using these TENGs in wearable electronics.^[^ ^52^, ^53^, ^54^ ^]^
- Due to its outstanding electron attraction ability, PDMS is widely adopted as the common triboelectric layer to acquire negative charges in TENGs.^[^ ^55^, ^56^, ^57^ ^]^ The PDMS films have also been considered the ideal choice of dielectric for biocompatible and deformable sensors. However, plain PDMS film‐based TENGs could provide a high output voltage under large external stimulation. Still, they often show a relatively low sensitivity by the reason of limited contact areas, which may not be suitable for human physiological sensing applications.^[^ ^1^, ^45^, ^58^ ^]^

In recent years, hydrogels and ionic hydrogels have attracted increasing interest in flexible electrode applications due to their ultrahigh‐stretchability, natural biocompatibility, and excellent transparency in the visible spectrum.^[^ ^59^ ^]^ DN hydrogel consists of two kinds of cross‐linked polymer networks: three‐dimensional hydrophilic polymers containing amounts of water.^[^ ^60^ ^]^ Therefore, the DN hydrogel demonstrates liquid‐like flexibility, solid‐like mechanical properties, and ionic characteristics.^[^ ^61^, ^62^ ^]^ Hydrogels have been employed to fabricate various soft electronics, including sensors (e.g., strains,^[^ ^63^ ^]^ gases,^[^ ^64^ ^]^ tactile,^[^ ^65^ ^]^ and temperature^[^ ^12^ ^]^ sensors), healthcare monitoring devices,^[^ ^66^ ^]^ and electronic skins based on TENG technology.^[^ ^65^, ^67^, ^68^ ^]^ However, hydrogels have two significant problems in real applications. One is the severe dehydration phenomenon when hydrogels are exposed to the external environment at a high temperature, accelerating water evaporation. The other is the solidification effect at a very low temperature caused by the freezing of water, which will significantly deteriorate the mechanical properties of the hydrogels. Great efforts have been made to improve the performance of hydrogels, such as the construction of organo‐hydrogels,^[^ ^63^ ^]^ ionic hydrogels,^[^ ^62^, ^69^ ^]^ or CNT hydrogels,^[^ ^70^ ^]^ which provide the inspiration of the ionic DN hydrogel in this work.

Herein, an ionic DN hydrogel using a novel lithium bromide immersion treatment (LBIT) method is produced as both the triboelectric layer and flexible electrode, providing a promising solution to achieve hydrogel‐based TENG and address the major problems mentioned above. Functionalized polyacrylamide (Paam)/carrageenan DN hydrogel is used to realize ultrahigh‐stretchability, good transparency, and high conductivity. Moreover, to achieve its low freezing point and excellent hydration ability with complementary water, 50% LiBr saturated solution at room temperature(RT) is adopted to incorporate in the hydrogel via a facile immersion treatment strategy. As a result, the LBIT DN hydrogel displays a freezing point below −110 °C and an outstanding anti‐dehydrating ability, achieving good environmental tolerance of the hydrogel‐based TENG sensor. Unlike the bulk hydrogel‐based TENGs, which lack high sensitivity and good flexibility, thin‐film structured LBIT ionic DN hydrogel is fabricated and utilized in the tactile sensors to achieve a better performance.

Based on the thin‐film structured LBIT ionic DN hydrogel, two types of THSs are presented according to their different working principles, that is, double‐electrode triboelectric hydrogel sensor (DE‐THS) and single‐electrode triboelectric hydrogel sensor (SE‐THS). Both triboelectric surfaces of the DE‐THS and the SE‐THS are micro‐pyramid‐patterned. The output performance can be significantly enhanced with the proposed hydrogel sensors. The newly fabricated hydrogel sensors have the following outstanding merits:

- The proposed hydrogel can be synthesized using a one‐pot polymerization method which is convenient for mass production. With a simple spin‐coating and bonding process, both the DE‐THS and the SE‐THS can be fabricated conveniently with an ultra‐thin thickness (i.e., 640 µm for DE‐THS and 400 µm for SE‐THS), giving rise to remarkable flexibility and high transparency (≈85%) of the hydrogel sensors.
- For the first time, the micro‐pyramid structures are patterned on the surface of the ionic DN hydrogel. The micro‐pyramid‐patterned THS largely increase the contact area of the self‐powered sensor, significantly increasing the sensitivity (up to 45.97 mV Pa^−1^) and lowering the detection limit (down to 50 Pa), which makes it suitable for wearable electronics applications.
- Multi‐functional LBIT ionic DN hydrogel is used as both the flexible electrode and triboelectric layer, achieving excellent self‐power functions, long‐term anti‐freezing and anti‐dehydrating properties, excellent environmental tolerance (−20 to 60 °C), and an instantaneous peak power density of 20 µW cm^−2^, which provides reliable electrical outputs for material type recognition and slight human motion detection.
- By integrating the signal acquisition/process circuit with micro‐pyramid‐patterned THS structures, the THS with an excellent self‐power sensing ability is utilized as a switching button to control electric appliances and robotic hand by monitoring human finger gestures, offering new insights of realizing single self‐powered THS for wearable and multi‐functional electronic applications.

## 2 Results and Discussion

**Figure 1:**
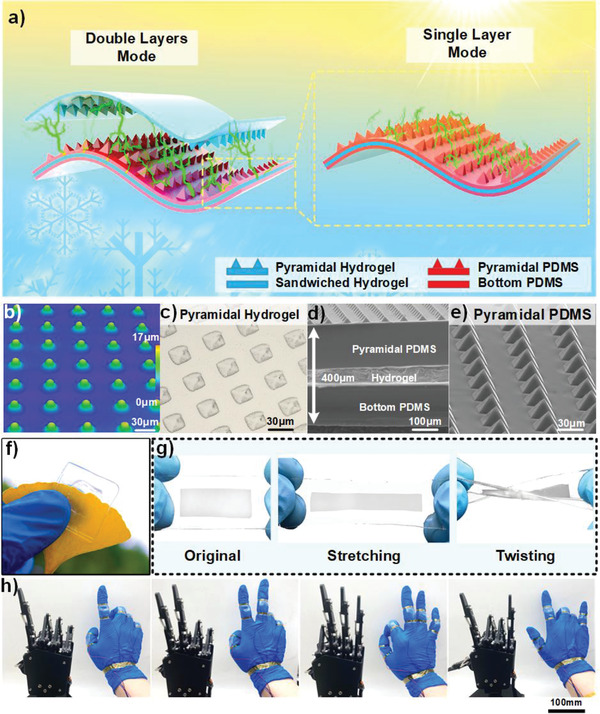
Structure design of the DE‐THS and SE‐THS: a) Schematic diagram showing the detailed structures, anti‐dehydrating and anti‐freezing properties of the ultra‐deformable DE‐THS and SE‐THS. b,c) CLSM image and optical microscope photograph of the micro‐pyramid structure on hydrogel surface. d) Cross‐section SEM of the SE‐THS. e) SEM image of the micro‐pyramid structure on the PDMS. f) Optical image of the DE‐THS. g) Optical images of original, stretching, and twisting of SE‐THS. h) Demonstrations of the robotic hand gestures corresponding to four different hand motions based on the SE‐THS.

In this research, the self‐powered functions of LBIT hydrogel sensors are based on the triboelectrification mechanism, which can continuously convert the tiny forces into electric signals within a wide temperature range. As shown in **Figure** **1**a, the basic architecture of DE‐THS and the SE‐THS are assembled by combining PDMS and hydrogel membranes to achieve a highly transparent, ultra‐flexible, and thin‐film structure. For the DE‐THS on the left‐hand side in Figure 1a, the LBIT hydrogel with micro‐pyramid structures is adopted as the top electrode and the friction layer to enable the device's higher flexibility and environmental tolerance. The micro‐pyramid structures on the hydrogel's surface can significantly increase the friction area during the triboelectrification process and thus improve the power output performance of the devices. The micro‐pyramid structures on the hydrogel surface can be clearly observed from the images by a confocal laser scanning microscope (CLSM) and an optical microscope, as shown in Figure 1b,c, respectively. The lower sandwiched structure consists of two layers of PDMS films and an encapsulated LBIT hydrogel. The total thickness is only 400 µm, as seen from the scanning electron microscope (SEM) image (Figure 1d). The upper PDMS film with the micro‐pyramid structures, as shown in the SEM image of Figure 1e, is oppositely adhered to the top of the hydrogel electrode for enhancing the triboelectric effect. The micro‐pyramid‐patterned PDMS film is applied as the electrification layer to generate triboelectric charges with the contacted objects of the SE‐THS on the right‐hand side.

Fabrication processes of both the SE‐THS and the DE‐THS are detailed in Figure S1, Supporting Information. Figure S2a,b, Supporting Information, show that the thicknesses of the DE‐THS and the SE‐THS are 640 µm and 400 µm, respectively, demonstrating ultra‐thin multilayer hydrogel structures. Figure S2c, Supporting Information, exhibits the transparency properties of both the DE‐THS and the SE‐THS hydrogel films. The obtained transmittance spectra indicates that the DE‐THS's transparency is slightly lower than those of thin‐film hydrogel and SE‐THS but still remains high level, for example, over 85% transmittance within the wavelength range between 400 to 800 nm. Owing to the excellent optical transparency of the DE‐THS, a leaf beneath the sensor could be clearly observed without any distortion (Figure 1f). Figure 1g demonstrates that the SE‐THS could withstand various deformations, including stretching and twisting, with outstanding flexibility. Five SE‐THSs are attached to the human knuckles to monitor the finger bending motions, which could be used to control the robotic hand. Figure 1h demonstrates the gestures of the human hand and the corresponding movement responses of the robotic hand.

A one‐pot polymerization method is introduced to synthesize the ultra‐stretchable and transparent DN hydrogel, and the synthesis method is described in Figure S3, Supporting Information, and Experiment Section. After that, 50 wt% LiBr solution is introduced in the pristine hydrogel using an immersion treatment method to avoid dehydrating and freezing because free water molecules in the pristine hydrogel can evaporate or freeze naturally due to the ambient temperature variation. Whereas after the LiBr solution immersion treatment, the water molecules in hydrogels can bond with Li^+^ and Br^−^ ions, and the higher degree of Li^+^ and Br^−^ ionic hydration is, the stronger bonding strength with H^+^ and O^−^ in water and the more bonded water molecules will be.

**Figure 2:**
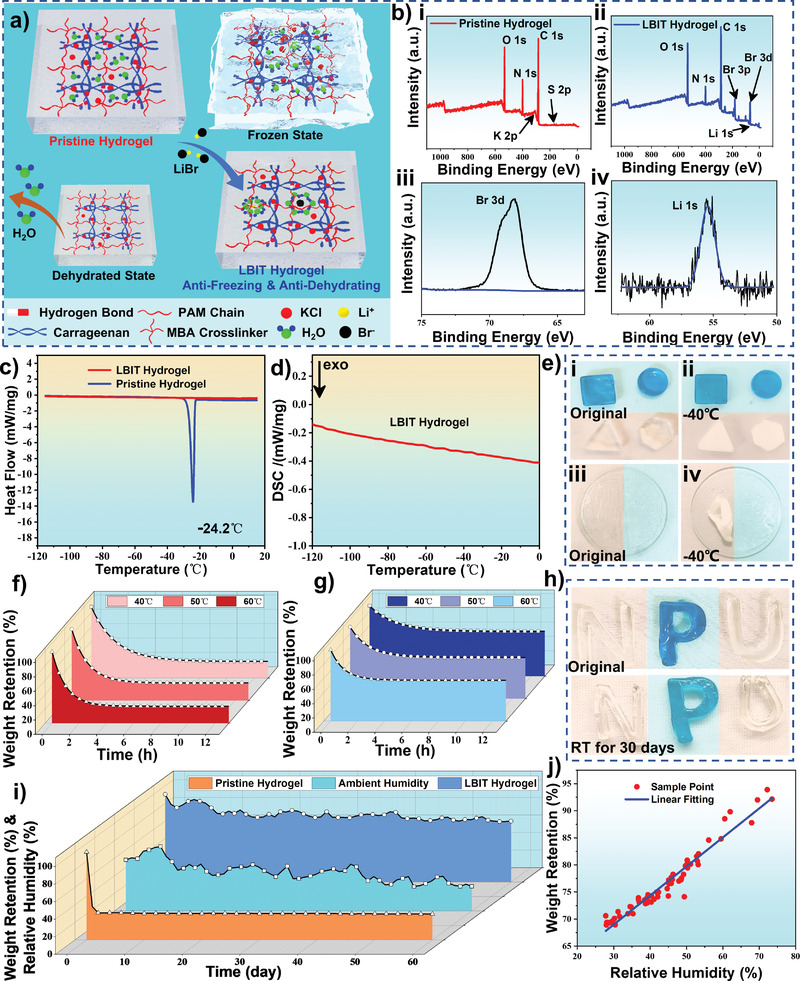
Anti‐dehydrating and anti‐freezing properties of the hydrogel: a) Schematic illustration shows the molecular architecture, anti‐dehydrating and anti‐freezing characteristics of pristine hydrogel and LiBr immersion treated (LBIT) hydrogel. b) XPS spectra of i) pristine hydrogel, ii) LBIT hydrogel, iii) Br 3d, and iv) Li 1s, respectively. c,d) DSC measurement of the pristine hydrogel and LBIT hydrogel. e) Anti‐freezing properties of the pristine hydrogel (in the orange region) and the LBIT hydrogel (marked in the blue region) in different shapes stored at −40 °C for 30 days. f) Comparison of weight retention of the pristine hydrogel under 40, 50, and 60 °C at 30% RH. g) Comparison of the anti‐dehydrating properties of LBIT hydrogel under 40, 50 and 60 °C at 30% RH. h) Anti‐dehydrating property of the pristine hydrogel (N‐shaped and U‐shaped in the orange region) and LBIT hydrogel (P‐shaped and marked in the blue region) after exposure at RT for 30 days. i) Weight change of pristine hydrogel and LBIT hydrogel after being kept at RT for 60 days and the ambient humidity variation during this period. j) Relationship between the weight retention and relative humidity of the LBIT hydrogel.

In consequence, the LBIT hydrogel achieves excellent anti‐dehydration and anti‐freezing properties (**Figure** **2**a). Based on elemental analysis results from X‐ray photoelectron spectroscopy (XPS) shown in Figure 2b‐i,ii, high content of C (over 50%), medium content of O (about 20%), and little contents of N (near 9%) could be found in the pristine and LBIT hydrogels (see Table S1, Supporting Information). Elements of Li and Br could also be detected in the XPS spectrum of LBIT hydrogel, which verifies the strong ionic hydration effect of LiBr (Figure 2b‐iii,iv).

To investigate the anti‐freezing properties of LBIT hydrogel and pristine hydrogel, their differential scanning calorimetry (DSC) spectra are measured within a temperature range from −110 to 20 °C, and the obtained results are shown in Figure 2c. The sharp peak in the spectrum at around −24.2 °C are linked to the freezing point of the pristine hydrogel. In contrast, there is no apparent DSC peak found on the spectrum of the LBIT hydrogel (Figure 2d), verifying its outstanding anti‐freezing ability.

LBIT hydrogel with a polygon shape (marked in the blue region) and pristine hydrogel (in the orange region) were stored at −40 °C for 30 days. The pristine hydrogel is frozen completely, whereas the LBIT hydrogel remains unchanged (Figure 2e‐i,ii). The membrane states (≈100 µm thick) of the pristine hydrogel film (in the orange area) were also stored at −40 °C for 30 days and became severely deformed and rigid. In contrast, the LBIT hydrogel film (with a similar thickness in the blue region) again remains unchanged (Figure 2e‐iii,iv), which can be attributed to the excellent low freezing point and strong hydration properties of the LBIT hydrogel. Further experiments about the anti‐freezing properties of the LBIT hydrogel under extremely low temperature (−78.5 °C) were conducted. The shape evolutions of LBIT hydrogel prototypes with different LiBr percolation percentages of 15 wt%, 35 wt%, and 50 wt% were recorded for one hour under the extremely low‐temperature environment (−78.5 °C). As is shown in Figure S4a, Supporting Information, the LBIT hydrogel prototypes with LiBr percolation percentages of 15 wt% and 35 wt% completely frozen after storage at −78.5 °C. In contrast, the 50 wt% LiBr treated hydrogels still keeps in the gel state without any slurry, indicating remarkable freezing and dehydration tolerance abilities of the proposed 50 wt% LBIT hydrogel material.

In‐depth evaluations for the anti‐dehydrating effect of the LBIT hydrogel were also conducted. The evolutions of weight retention for both the LBIT and the pristine hydrogels are recorded under different temperatures, and three groups of each sample were kept in an oven at 40 °C, 50 °C, and 60 °C, respectively. Due to the significant evaporation of water inside the gel, the weight of pristine hydrogel decreased rapidly and dropped down to 25% within the first 8 h (Figure 2f). Whereas the weights of LBIT hydrogel maintain around 65%, 60%, and 58% of their original weights after being kept at 40 °C, 50 °C, and 60 °C, respectively (Figure 2g). A long‐term dehydrating test was also implemented. After 30 days of storage at RT under the ambient condition of our laboratory (25 °C, 60% relative humidity (RH)), the size of the pristine hydrogel (N‐shaped and U‐shaped in the orange region) becomes much smaller and rigid, indicating its severe shrinkage (Figure 2h). In contrast, the LBIT hydrogel (P‐shaped and marked in the blue region) almost keeps its original state, which is mainly due to the strong hydration ability of Li and Br. A vacuum‐freeze test was also performed for these two types of samples. As shown in Figure S4c,d, Supporting Information, the LBIT hydrogel is almost unchanged after one month, whereas the pristine hydrogel is frozen within 1 min and significantly dehydrated after one month.

Further experiments were conducted to investigate the humid air absorption effect of the two types of hydrogels. Figure 2i shows the weight changes of both the pristine hydrogel and the LBIT hydrogel while being stored at RT for 60 days. The ambient humidity variation levels were recorded during this period. The weight variations of the LBIT hydrogel with the ambient humidity level were also measured, and the data are shown in Figure 2j, which have a noticeable positive correlation with the ambient humilities due to the humid air absorption effect. The pristine hydrogel dried thoroughly within three days. On the contrary, the LBIT hydrogel almost maintained its original state, mainly due to the LiBr's strong hydration properties. Structural optimization was calculated by using b3lyp 6–311g++ basis functions in Gaussian 09. As is demonstrated in Figure S5a,b, Supporting Information, the Li+ and Br− ions can form the molecular clusters with the neighboring water elements due to the strong ion‐dipole interaction between ions and molecules, suppressing the formation of hydrogen bonds between the water molecules. As a result, the LBIT hydrogel exhibits an extremely low freezing point as well as an excellent hydration effect. This makes the LBIT hydrogel a suitable candidate for humidity sensing, which will be deliberated in the following sections.

**Figure 3:**
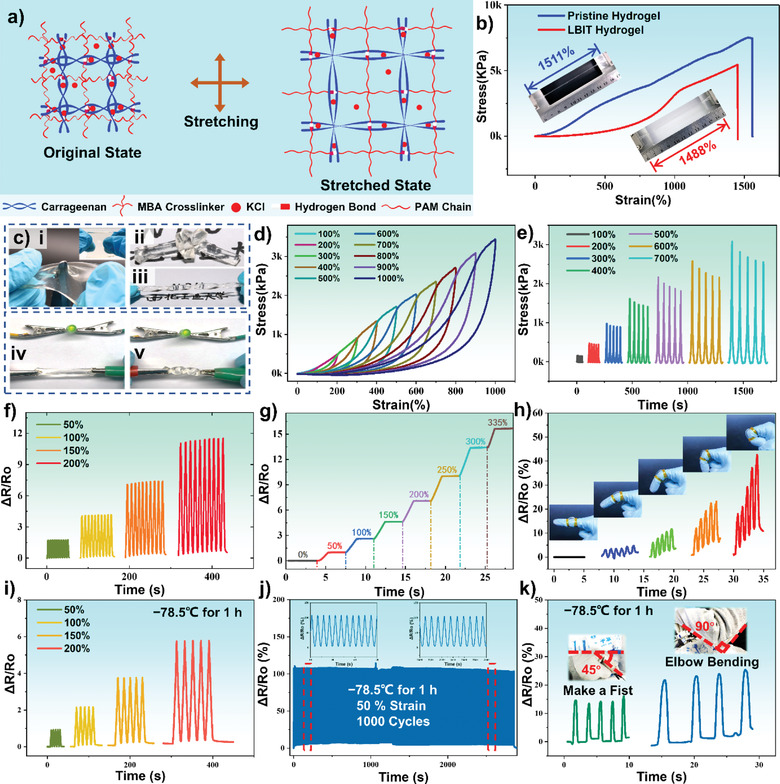
Electromechanical properties of the double‐network LBIT hydrogel: a) Schematic illustration shows the double‐network architecture of the PAM chain and the Carrageenan chain in the hydrogel. b) Strain–stress curves of pristine hydrogel and LBIT hydrogel. c) Mechanical deformability and conductivity of the LBIT hydrogel. d) Stretching and releasing tests from 100% to 1000% of LBIT hydrogel. e) Cyclic test of the LBIT hydrogel under 100% to 700% stretching states. f) Comparison of dynamic resistance of 10 cycles between different deformations sequentially, including 50%, 100%, 150%, 200% strain. g) Relative resistance variation versus strains from 0% to 335%. h) Real‐time responses of the LBIT hydrogel to different bending states when attached to the index finger. i) The resistance variations (Δ*R*/*R*_0_) at cyclic strains after storage at the temperature of −78.5 °C. j) The long‐term durability test of the LBIT hydrogel after being stored at −78.5°C for 1 h. k) Resistance variation curves of the LBIT hydrogel on the fist and the elbow of a human body at different bending angles.

Two types of interconnected networks are formed in the LBIT ionic DN hydrogel by the polymerizations of carrageenan and PAM chains. Therefore, the ionic DN hydrogel demonstrates excellent mechanical properties under loading stress, as schematically illustrated in **Figure** **3**a. To evaluate its mechanical properties, tensile tests were conducted on the fabricated hydrogels. Both the pristine hydrogel and LBIT hydrogel were stretched to their maximum elongations before breaking (insets of Figure 3b). The LBIT hydrogel shows similar stretchability (up to 1488%) as that of the pristine hydrogel (1511%), and the tensile strength of pristine hydrogel is a little higher than that of the LBIT hydrogel (Figure 3b).

The stretchability and recoverability of the LBIT hydrogel were further investigated with the strain from 100% to 1000%, and the obtained results are shown in Figure 3d. There are slight hysteresis loops found at each cycle, which is mainly due to the dynamic dissociations of the physical network chains and the hydrogen bonds. Moreover, the mechanical stability of the LBIT hydrogel was studied by stretching it for five cycles under a fixed strain from 100% to 700% (Figure 3e). Although there are slight decreases in the maximum stress values after each cycle, the cycling curves are generally entirely consistent during cycling deformation, demonstrating an excellent elastomer‐like behavior of the LBIT hydrogel due to its double‐network structure and cross‐linking interactions. It shows outstanding mechanical performance at different extreme deformation situations, including punching, knotting, and twisting (Figure 3c‐i–iii). For example, no apparent damage appears on the LBIT ionic DN hydrogel surface after it is punctured by a sharp knife, as demonstrated in the inset of Figure 3c‐i. It is worth noting that the LBIT hydrogel can still be stretched to 3.5 times from its original state even exposed to the ambient air for as long as two years, as demonstrated in Figure S5c, Supporting Information. The enhancement of the water‐retention capacity by LBIT hydrogel originates from the ion‐dipole interaction between the ions such as Li^+^, Br^−^, and H_2_O, significantly decreasing the dehydration of water molecules.

Moreover, attributing to its content of K^+^, Cl^−^, Li^+^, and Br^−^ ions, the LBIT hydrogel has the ability to maintain its conductivity under various types of deformations. As exhibited in Figure 3c‐iv,v, an LED is connected in series with the LBIT ionic DN hydrogel in a circuit. When the hydrogel is stretched or twisted, the LED remains its bright color without apparent degradation, demonstrating its excellent conductivity in complex deformation conditions.

Attributed to the strong hydration ability of LiBr, the LBIT hydrogel could not be freeze‐dried completely at −80 °C with a vacuum‐freeze treatment (Figure S4d, Supporting Information). Therefore, we use the freeze‐dried pristine hydrogel for the SEM characterization (Figure S4b, Supporting Information). The SEM image shows the highly porous structures of the hydrogel, and the interconnected porous structure provides a suitable pathway for the ions, which contributed to the excellent conductivity at different deformation conditions.

Furthermore, a strain sensor was fabricated using the LBIT ionic DN hydrogel, and its resistance changes at different strains were tested. The obtained results are shown in Figure 3f–g. The relative resistance change Δ*R*/*R*_0_ during the hydrogel deformation was calculated to evaluate the electromechanical properties of the strain sensor. *R*_0_ is the initial resistance of the hydrogel sensor, where Δ*R* is its resistance variation. As is shown in Figure 3f, there is slight variation of the Δ*R*/*R*_0_ values for the LBIT hydrogel in the ten cyclic tests at different strains (i.e., 50%, 100%, 150%, 200%), revealing the excellent electrical stability of the LBIT hydrogel. Dynamic resistance variations were further recorded when the LBIT hydrogel was deformed at a given strain for 2.5 s, and then the strain was increased and maintained at a higher value for another 2.5 s. This was repeated for the strain values from 0% to 335% gradually. The obtained results are shown in Figure 3g, revealing the excellent stability of the resistance values at a fixed strain for a few minutes.

Based on its excellent electric properties, a strip of the LBIT ionic DN hydrogel was attached to the finger joint of a person and utilized as a strain sensor to monitor the different bending states of the finger joint (Figure 3h). The relative resistance value shows significant changes in response to the different bending angles of the finger in real‐time. The larger the bending angle, the larger the resistance values change. Further experiments about the anti‐freezing properties of the LBIT hydrogel under extremely low temperature (−78.5 °C) were conducted. As is shown in Figure 3i, there is only a slight variation of the Δ*R*/*R*_0_ values for the LBIT hydrogel in the five cyclic tests at different strains (i.e., 50%, 100%, 150%, 200%), revealing the excellent electrical stability of the LBIT hydrogel under the extreme cold conditions. Moreover, the fabricated LBIT hydrogel also shows a repeatable and stable response during a continuous stretch‐release cyclic test with 50% strain for 1000 cycles after being stored at −78.5 °C for 1 h, as shown in Figure 3j. For wearable demonstrations, the LBIT hydrogel prototypes were further attached to the fist and the elbow of a human body at such a low temperature (Figure 3k). The relative resistance value shows significant real‐time changes in response to the different bending angles, demonstrating good anti‐freezing properties of the LBIT hydrogel for wearable applications in an ultra‐low temperature environment. These results clearly indicate that the strip‐shaped LBIT ionic DN hydrogel has excellent sensitivity and adaptability to monitor finger motions, demonstrating its good prospects for biomechanical applications.

**Figure 4:**
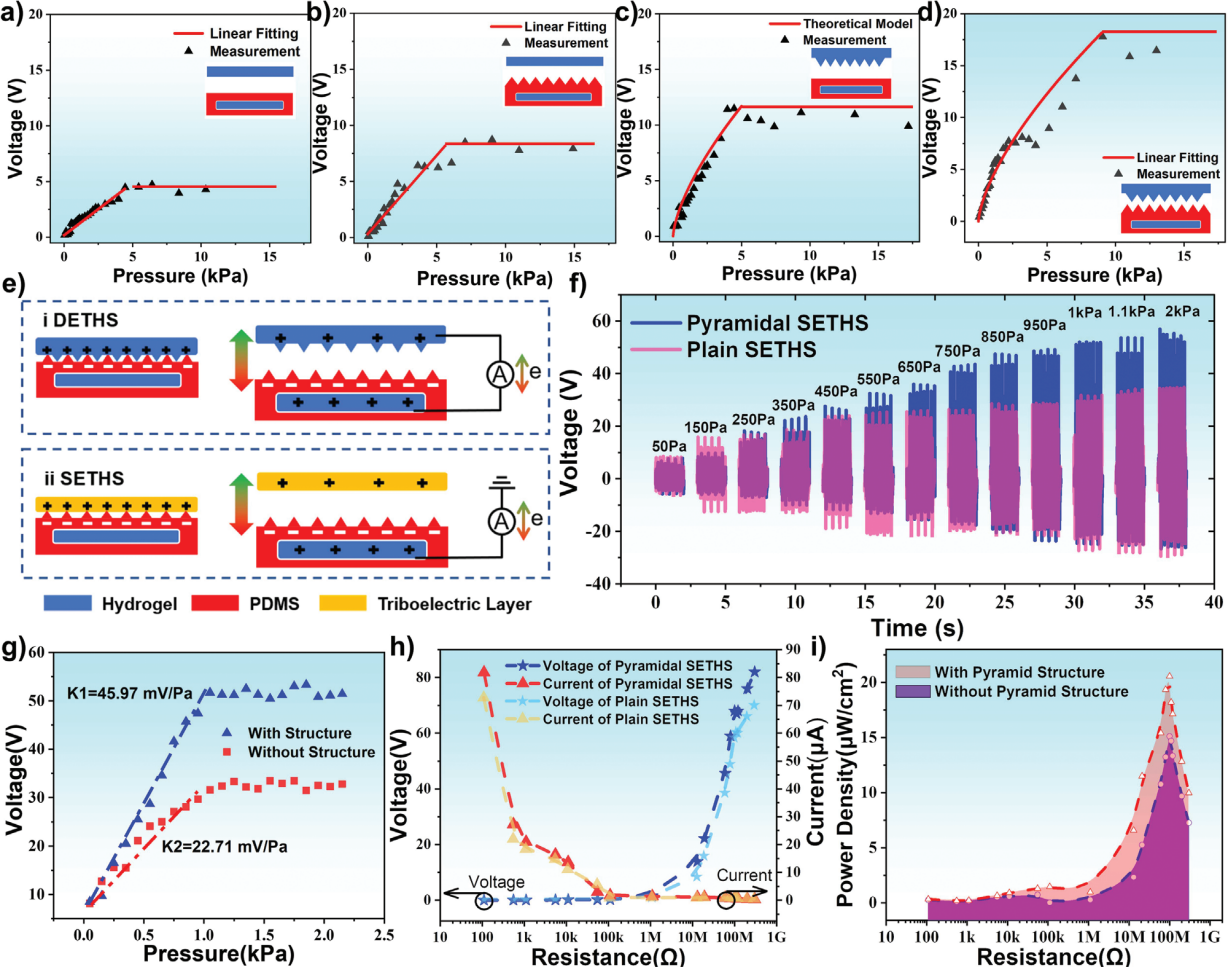
The basic principles, characterizations, and performances of DE‐THS and SE‐THS: Open‐circuit voltage variation under different pressures of four kinds of DE‐THS: a) Plain DE‐THS; b) DE‐THS with micro‐pyramid‐patterned PDMS and plain hydrogel; c) DE‐THS with micro‐pyramid‐patterned hydrogel and plain PDMS; d) DE‐THS with both micro‐pyramid‐patterned PDMS and hydrogel. e) Schematic illustration of the working principle of DE‐THS and SE‐THS. f) Voltage curves under different applied pressures of plain SE‐THS and micro‐pyramid‐patterned SE‐THS. g) Open‐circuit voltage variation under different pressures of plain SE‐THS and micro‐pyramid‐patterned SE‐THS. h) Dependence of the output on the external resistance load of plain SE‐THS and micro‐pyramid‐patterned SE‐THS. i) Dependence of the power density on the resistance of external load of plain SE‐THS and micro‐pyramid‐patterned SE‐THS.

The multilayered hydrogel structures were further prepared by a sequence of spin coating, molding, peeling off, and final assembly process and the fabrication method is demonstrated in Figure S1, Supporting Information. They were cut to the same size of 1.5 × 1.5 cm^2^ for characterization. Four different types of the DE‐THS devices were fabricated and characterized, including plain DE‐THS (**Figure** **4**a), DE‐THS with micro‐pyramid‐patterned PDMS and plain hydrogel (Figure 4b), micro‐pyramid‐patterned hydrogel and plain PDMS (Figure 4c), both micro‐pyramid‐patterned PDMS and hydrogel (Figure 4d). Obviously, the output voltages of all types of DE‐THSs increase with applied pressure initially but then reach a plateau of the maximum voltages. As shown in Figure 4a, the output voltage of the first DE‐THS sample (i.e., plain PDMS and plain hydrogel) increases with the applied pressure in the range of 20 Pa to 4 kPa, and the maximum output voltage of 4.5 V is obtained when the external pressure reaches its saturation pressure of 4.5 kPa. Figure 4b,c,d show that the saturation voltages is 8.5 V at a pressure of 7 kPa for the second DE‐THS (i.e., micro‐pyramid‐patterned PDMS and plain hydrogel), 12 V at 4.5 kPa for the third DE‐THS (i.e., plain PDMS and micro‐pyramid‐patterned hydrogel), 18V at 9 kPa for the fourth DE‐THS (i.e., micro‐pyramid‐patterned PDMS and micro‐pyramid‐patterned hydrogel). The presence of both micro‐pyramid‐patterned PDMS and micro‐pyramid‐patterned hydrogel significantly improves the sensitivity if compared to that of the plain device, mainly due to the effectively increased contact areas and enhanced triboelectric effect.

The operating principle of the DE‐THS is schematically illustrated in Figure 4e‐i. When the external force is applied to the DE‐THS, the top conductive hydrogel layer contacts with the bottom friction layer of the PDMS, generating electrical voltage signals due to electrostatic induction and triboelectrification effects. The triboelectric effect occurs when these two fiction layers get into contact with each other, and equivalent charges with the opposite polarities could be transferred between these two contact surfaces due to the different electron affinities. Compared to that of hydrogel, PDMS has a higher electron affinity and can acquire negative charges. Once these two friction layers are separated, the hydrogel electrode will generate positive charges due to electrostatic induction. As a result, electrons will be transported to the top hydrogel electrode at the same time. An electrostatic equilibrium is achieved when these two friction layers are separated to a certain distance, above which there is no further current in the external circuit. As the upper hydrogel electrode is pressed back to contact with the bottom electrode, the process is reversed, and the currents in the opposite direction could be generated.

A theoretical model is proposed to investigate the enhanced effect of micro‐pyramid‐patterned hydrogel on the performance of fabricated DE‐THS output voltage. Details of the equation derivation is shown in Section S1, Supporting Information. For the third DE‐THS, the output voltage can be expressed as:

(1) $$V_{\mathrm{OC}}=\frac{Q}{C}=\frac{2.36\sigma A_{\mathrm{device}\;\mathrm{total}}{\left(\frac{tp}{EL}\right)}^{\frac{2}{3}}}{\varepsilon A_{\mathrm{device}\;\mathrm{total}/t}}=2.36\left(\frac{\sigma t}{\varepsilon }\right){\left(\frac{tp}{EL}\right)}^{\frac{2}{3}}$$

where *V*_OC_ is the open‐circuit voltage determined by the total triboelectric charge (*Q*) and the parasitic capacitance of PDMS (*C*); *σ* is the surface charge density; *A*_device total_ is the total triboelectric area of the device; *ε* denotes the dielectric constant of the PDMS; *p* and *E* represent the applied pressure on the device and Young's modulus of the hydrogel, respectively; *t* is the thickness of the PDMS layer; *L* is the length of the bottom edge of the micro‐pyramid structure.

Based on Equation (1), *V*_OC_ increases when a larger pressure *p* is applied. Due to the elastic deformation of the micro‐pyramid structures, the contact area *A*_device total_ between the PDMS and hydrogel has been increased significantly compared with that of the plain DE‐THS. When the micro‐pyramid structures on the hydrogel surface are fully deformed, *A*_device total_ will reach its maximum value. Thus the triboelectric effect reaches its saturated state, and the voltage will not be further increased. Therefore, the output performances of the micro‐pyramid‐patterned DE‐THS are in good agreement with the theoretical results (Figure 4c; Text S1, Supporting Information).

It should be addressed that the output voltage of the fourth DE‐THS (i.e., micro‐pyramid‐patterned PDMS and micro‐pyramid‐patterned hydrogel) continuously increases with the increase of pressure from 20 Pa to ≈9 kPa. A maximum output voltage of 18 V is obtained, which shows the highest voltage value and saturation pressure among four types of DE‐THSs. The dramatic output increase of the DE‐THS with both the pyramidal PDMS and hydrogel is attributed to the following factors: i) The capacitance changes in the contact‐deform‐separate process are significantly enhanced due to the existence of air gaps and the larger dielectric constant of the PDMS. ii) Both the PDMS and hydrogel are elastomers, therefore the micro‐pyramid structures can be compressed significantly, which causes a considerably increased contact area between two materials and thus more electrons generated during the contact triboelectrification process. iii) The 3D morphologies on the surface of both triboelectric layers significantly reduce the adhesion effect, thus making it easier for two contact surfaces to separate, increasing the dipole moment and enhancing the output performances.

Due to the different electron acquisition abilities of the PDMS and the hydrogel, the SE‐THS has been further investigated in this work. Figure 4e‐ii illustrates the basic operating principle of the SE‐THS, which is based on the coupling of the triboelectric effect and electrostatic induction. A chemigum layer is used as the lower charge affinity material to contact the PDMS layer of the SE‐THS. Contact electrification occurs on the interface when the chemigum layer is pressed onto the friction layer of PDMS, and equally opposite charges are generated on both sides. Since PDMS is easier to gain electrons, electrons will be transferred to PDMS from the chemigum layer. Once these two friction layers are separated, the electrons will flow back from the hydrogel electrode to balance the negative charges on PDMS. After the electrostatic equilibrium state is reached, there is no net current exists in the circuit. Once the two layers are contacted again, current in the opposite direction is detected due to the flow back of electrons. Accordingly, a cyclic alternating current output is generated by applying repeated contact–separation processes.

To further investigate the output performance of SE‐THS, two types of SE‐THS are fabricated, that is, plain SE‐THS and micro‐pyramid‐patterned SE‐THS. Similar experiments used in the tests of DE‐THS have been implemented here to characterize the sensitivity of the fabricated SE‐THS for the self‐powered pressure sensing applications. Figure 4f shows the obtained output voltage waveforms of the plain SE‐THS and the micro‐pyramid‐patterned SE‐THS, indicating the saturate pressure points of the output performances are at around 1000 Pa and 1500 Pa, respectively. The relationship between the output voltage and applied pressure of two kinds of devices is depicted in Figure 4g. Clearly, the open‐circuit voltages of both devices growth initially with the increased external pressure. However, with the further increase of the pressure, the output voltage approaches a saturation value, which shows a similar trend with that of DE‐THS. The voltage output of the micro‐pyramid‐patterned SE‐THS shows a much higher value compared with that of the plain SE‐THS. It is worth mentioning that the micro‐pyramid‐patterned SE‐THS achieves a sensitivity of 45.97 mV Pa^−1^, which is twice as much as the 22.71 mV Pa^−1^ of the unstructured SE‐THS. This is mainly attributed to the presence of the proposed micro‐pyramid structures, as explained before.

Furthermore, owing to the considerably large electron affinity difference between the chemigum layer and PDMS, the output voltage of the SE‐THS is much higher than that of DE‐THS, mainly because the chemigum quickly loses electrons than the hydrogel in the triboelectric process. To find the optimal load condition of the SE‐THS, the changes of output currents and voltages as well as the external resistance values are studied, and the results are shown in Figure 4h. The open‐circuit voltage and short‐current of micro‐pyramid‐patterned SE‐THS around 82 V and 83 µA can be obtained, respectively. The micro‐pyramid‐patterned SE‐THS exhibits a much larger output compared with those of the plain SE‐THS in almost all external resistance range. Figure 4i shows that both the devices achieve their maximum power densities by using around 100 MΩ resistance load. Moreover, the micro‐pyramid‐patterned SE‐THS has a maximum peak power density of ≈20 µW cm^−2^ under 1500 Pa, which is higher than that of the plain device (15 µW cm^−2^). Table S2, Supporting Information, summarizes the comparisons of the‐state‐of‐art thin‐film flexible sensors with respect to operating principles, transparency, thickness, self‐power operation, temperature tolerance, and pressure sensitivity. It is shown that the proposed micro‐pyramid‐patterned hydrogel sensor has demonstrated outstanding performance with excellent flexibility, high transparency, good sensitivity, self‐powered operation, and large temperature tolerance.

**Figure 5:**
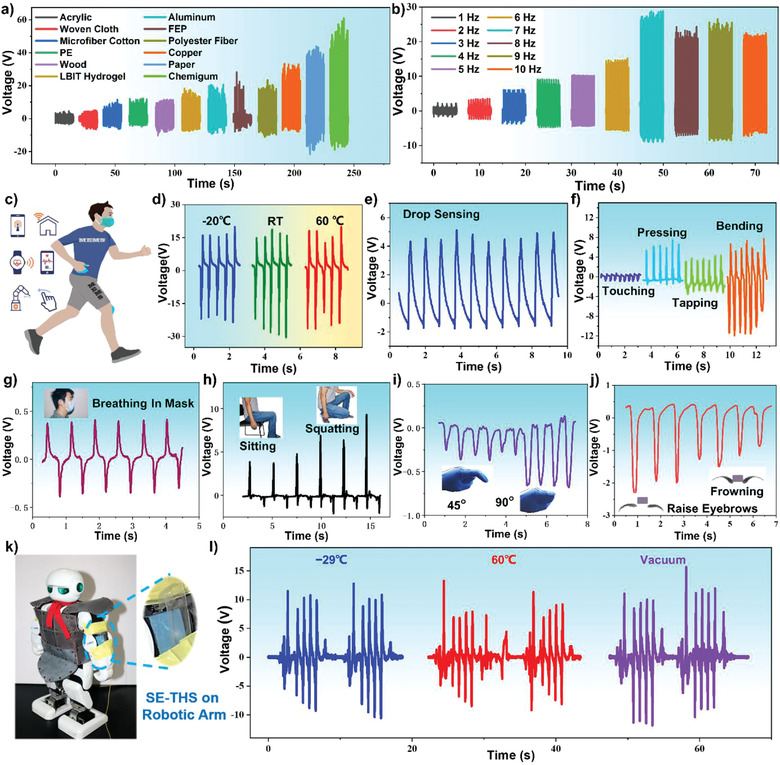
Diversified applications of DE‐THS and SE‐THS: a) Open‐circuit voltage of the SE‐THS under contact‐separation motion with different materials at 1 Hz by finger tapping; b) The open‐circuit voltage variation of SE‐THS at various applied frequencies by vibration table; c) Schematic diagram of the applications of the proposed SE‐THS: IoT, healthcare monitoring and robot control; d) Output voltage of the SE‐THS at different temperatures by finger tapping; e) Open circuit voltage of the SE‐THS in water drop sensing; f) Output signals of DE‐THS under touching, pressing, tapping, and bending; Applications of DE‐THS and SE‐THS on human motion detection: g) Eyebrow movement; h) Breathing monitor; i) Knee joint motion; j) Knuckle movement; k) Attach the SE‐THS onto the robotic arm to detect the elbow joint movements; l) The recorded output waveforms under the temperatures of −29 and 60 °C and in the room‐temperature vacuum conditions.

The micro‐pyramid‐patterned SE‐THS is further investigated for the identification of the most suitable materials for this application. Twelve common materials are utilized as the different triboelectric layers, including acrylic, woven cloth, microfiber cotton, polyethylene (PE), wood, LBIT hydrogel, aluminum, fluorinated ethylene propylene (FEP), polyester fiber, copper, paper, and chemigum. As shown in **Figure** **5**a, the obtained peak voltages of SE‐THS using different materials are significantly different. The output performance order of each material is arranged from left to right in Figure 5a, which is just according to the quantified triboelectric series,^[^ ^71^ ^]^ demonstrating its effectiveness and great potential in object and material recognition application scenarios. Moreover, when the SE‐THS contacts with the LBIT hydrogel layer (DE‐THS architecture), the peak output voltage reaches ≈18 V, indicating the slight polarity difference between the hydrogel and PDMS. The chemigum maintains the highest output voltage of over 50 V among the 12 different materials. As a result, SE‐THS with a chemigum layer is used in further tests due to its larger output voltage.

In addition, the effect of stimulation frequency on the device's performance is also investigated, and the result is shown in Figure 5b. With the increase of the stimulation frequency from 1 Hz to 7 Hz, the output voltages increase significantly from 2.5V to its maximum value of 25 V, due to the changed contact status between the SE‐THS device and the contacted object (chemigum is utilized in the test). The device and the chemigum material get full contact with each other at the frequency of 7 Hz, thus reaching its maximum performance of triboelectrification. However, due to the inherent mechanical limitation of the vibration system, the amplitude decreases slightly when the frequency further increases. Therefore, it is found that the output voltage of the SE‐THS could not monotonically increase as the frequency grows in the frequency range of 7–10 Hz.

Figure S6, Supporting Information, shows the electrical properties of the SE‐THS characterized by the finger‐tapping stimulation and the shaker tapping excitation at a constant frequency of 10 Hz. With the simple finger tapping stimulation, the transferred charge is around 4.6 nC with a considerable small area of 1.5 × 1.5 cm^2^, as depicted in Figure S6a, Supporting Information. Therefore, the experimental result of the transferred charge (*σ*) can be figured out up to 2.04 nC cm^−2^. It is smaller than the theoretical result (*σ*_0_) of 5.75 nC cm^−2^ according to the charge density expression *σ*_0_ *= ε*_0_ *ε*_r_ *Vd* ^−1^, where *ε*_0_ is the dielectric constant of vacuum, *ε*_r_ represents the relative dielectric constant of PDMS, *V* stands for the surface potential of PDMS, and *d* is the thickness of PDMS layer. It mainly attributes to the net charge density in the dielectric layer and the decay of the electric field in the ambient condition. The response and recovery curves as a function of time are shown in Figure S6b, Supporting Information. The fabricated SE‐THS has a fast response time of 17 ms and a recovery time of 23 ms during press and release period. Figure S6c,d, Supporting Information, show the output voltage responses and the enlarged views of the fabricated SE‐THS at a constant excitation frequency of 10 Hz by a mechanical shaker, respectively. The SE‐THS device shows excellent durability and the output voltage signal remains steady after 36 000 cycles. No apparent structural damage is observed during this period.

As shown in Figure 5c, the proposed THS is attached to human skins or assembled with wearable devices for the demonstration of various applications in the internet of things, healthcare monitoring, and robotic hand control. Considering the typical working conditions for using these devices, the ambient temperature in the fields usually varies from −20 °C to 60 °C. Therefore, the SE‐THS is tested at −20 °C, RT and 60 °C, respectively, and the obtained results are shown in Figure 5d. It has been confirmed that SE‐THS can perform effectively at these temperatures and voltage output of ≈16 V is generated when a simple finger pressing is applied. What is more, there is no significant drift of the outputs when the temperature changes over a wide range from −20 °C to 60 °C. Characterization results of the DE‐THS samples performed in the same testing environment that can be found in Figure S7, Supporting Information, which also demonstrates the good performance and stability of the DE‐THS at different temperatures.

The output voltages of the DE‐THS under different RH levels were further measured, and the obtained results are shown in Figure S8a, Supporting Information. When the RH of the environment is decreased from 90% to 40%, the peak voltage is gradually increased from 2.4 V to 3.5 V accordingly. This is mainly attributed to the excellent hydration ability of the upper hydrogel electrode/triboelectric layer in the DE‐THS. As the hydrogel layer continuously absorbs the moisture in the air, the triboelectrification effect between the hydrogel and PDMS will be weakened, leading to a decreased output voltage. The sensitivity of the DE‐THS to the humidity level is further calculated. Based on Figure S8b, Supporting Information, the DE‐THS has an RH sensitivity of ≈−17 mV 1% RH^−1^ within the RH level between 40% and 90%. Hence, the device has the potential for measuring the environment humidity in the ambient.

As the thickness of the fabricated SE‐THS can be reduced as thin as 400 µm, it can be applied to detect extremely small movements or minor stimulations. Figure 5e demonstrates the output voltage responses of the SE‐THS device when the stream of water droplets continuously flow on the device's surface, and an output peak voltage of 5 V with regular waveform can be detected during water droplet flowing. Figure S9a, Supporting Information, shows the self‐power generation principle during the water‐PDMS contact electrification process. When the droplets contact the micro‐pyramid‐patterned PDMS, triboelectrification happens on the interface between the liquid and the PDMS layer. The micro‐pyramid‐patterned PDMS will acquire the negative charges when the positively charged droplets flow away, leading to the alternative current between the inside hydrogel electrode and the ground. It is also found that with the increase of impinging droplets number, the surface potential on the original PDMS surface increases gradually and finally reaches around a stable value of −500 V (Figure S9b, Supporting Information). Figure S9c, Supporting Information, shows the dependence of the maximum output voltage on the height of the droplet. When the droplets (∼80 µL) are released from different heights ranging from 6.5 cm to 20 cm, the open‐circuit voltages vary correspondingly. It could be observed clearly that the output voltage increases with the growth of the height initially and then obtain the highest value of 26.9 V approximately, providing the potential for the self‐powered waterdrop speed detecting ability. Figure 5f shows the output signals of the DE‐THS device under multiple activations or deformations, including touching, pressing, tapping, and bending of the device. These are the four most common hand movements in our daily lives. The excitation force, frequency and compression state of the four stimulations are quite different. Take the touching motion as an example, only the index finger at the frequency of 2 Hz is applied to the DE‐THS to simulate the state of the fabricated device being touched as a tactile sensor. These experimental results demonstrate the capability of using the DE‐THS device to monitor human body movements.

The DE‐THS was further attached to a face mask, knee, finger knuckles, and eyebrows of an operator, as shown in Figure 5g–j, respectively, in order to demonstrate the detection of the operator's motions. Figure 5g reflects the integration of the DE‐THS into the inside of a face mask for potential breath sensing purposes. It is clearly seen that an output signal around 0.5 V with approximately sinusoidal waveforms is detected, with the volunteer's breathing rate around 1.5 Hz. When attached to the operator's knee, the dynamic output signals of the DE‐THS device can be obtained and perfectly match to different bending movements accordingly (Figure 5h). Figure 5i,j show the acquired signals from the DE‐THS device by the slight movements of finger knuckles and eyebrows, respectively. As shown in Figure 5i, the DE‐THS device responds well to the different bending movements of the finger knuckle. The output signal of the device at a bending angle of 90° is about 1.8 times that of 45°. As shown in Figure 5j, by attaching the SE‐THS between the operator's eyebrows, the eyebrow‐raising or frowning movements have generated different amplitudes of voltage signals from the device. The SE‐THS was further attached to the robotic arm to detect the activities of the elbow joint (Figure 5k). The output waveforms are also recorded at temperatures of −29 °C and 60 °C, and in room‐temperature vacuum conditions (−0.1 MPa). As is demonstrated in Figure 5l, the SE‐THS could respond well to press‐up movements and shows no apparent decline under the extreme environments of −29 °C, 60 °C, and even in the vacuum conditions, demonstrating the good application potential in the field of harsh environments.

**Figure 6:**
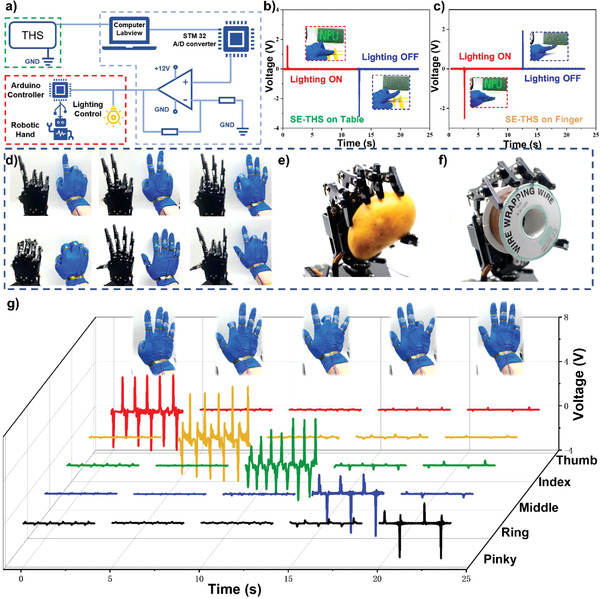
The implementation of SE‐THS for interactive human–machine interfaces application: a) Schematic diagram of the robot hand control and light control system; b) Output signal of SE‐THS attached to the table when serving as a light switch; c) Output signal of SE‐THS attached to the knuckles when serving as a light switch; d) The photographs of human hand gestures and corresponding robot hand motions; e) The photograph of grabbing a mango by robot hand; f) The photograph of grabbing a coil by robot hand; g) Generated outputs from five sensors: thumb, index finger, middle finger, ring finger, and pinky finger when fingers move successively.

A human–machine interface was further established to realize the functions of button switch and robotic hand control via the intuitionistic signal‐process methods. **Figure** **6**a illustrates the system‐level overview of the robot and light control system. The whole testing system contains of three main parts: 1) The THS sensors that are utilized to detect the movements of the finger joint. 2) The signal acquisition and processing system consists of a signal acquisition module, threshold value comparison module, signal amplifier module, and driving control module. 3) The controlled terminals consist of robotic hands or LEDs. Once the THS device generates the voltage signal, the output will be received by an Ni DAQ card (National Instrument Corporation). Peak voltage is collected and converted into a digital signal through a LabVIEW platform. The details of the software are shown in Figure S10, Supporting Information. Then, the pre‐set threshold value comparison function will process the voltage signals into five binary signals (0/1). These signals will be processed by STM 32 amplifier module and then used to drive servo motors, which will directly drive the robotic hands to imitate the human finger gestures. A customized power amplifier is utilized to magnify the electric appliance's direct current before applying it to the devices such as an LED bulb and a robotic servo.

The proposed SE‐THS has successfully demonstrated to control a button switch to operate the LED bulk for the light control system. As shown in Figure 6b, the SE‐THS is used as the button switch. When the operator presses the SE‐THS, the positive current generated by the contact gesture is processed by the machine interface system to switch on the LEDs. Once the pressure is released from the SE‐THS, the LED lights turn off immediately. Figure 6c shows the SE‐THS attached to the fingertip to touch an object, which can also be used as an intelligent switch. The dynamic processes for the SE‐THS's controlling the button switch in these two different modes can be found in Video S1, Supporting Information.

We further explore the possibility of detecting human finger movements that can be applied for robot hand control. Five SE‐THSs are carefully fastened by the polyimide tape on the second knuckles of each finger, providing the maximum bending angle of common hand gestures. There exist tiny gaps of around 1 mm between the SE‐THS and the chemigum gloves when the fingers are naturally straightened. Once the finger is bent, the SE‐THS will contact the bottom chemigum layer to generate the voltage signal due to the basic triboelectric working principle. When the finger returns to the straight state, the opposite signal will be produced. The voltage pulses generated by the SE‐THSs are utilized in five signal channels and further processed by a human–machine interface, which can drive five servos to control the robot hand and mimic the motions of the operator's hand. Figure 6d demonstrates that the robot hand can accurately imitate various gestures of the human hand. The dynamic movements can be found in the supplementary material Video S2, Supporting Information.

Figure 6e,f demonstrate that the robotic hands can be driven to grab a mango and a coil under the control of the SE‐THS, respectively. When we made multiple gestures by moving the finger knuckles, the generated signals of five SE‐THSs show quite different results, as shown in Figure 6g, which proves the excellent sensitivity of the SE‐THS to detect finger knuckle movements. The supplementary material Video S3, Supporting Information, indicates that the SE‐THSs attached to the multiple fingertips have no interference of signals among each other and also without any apparent signal delays. Combined with the human–machine interface system, the SE‐THS will indeed have promising prospects in surgery systems and manufacturing industries under harsh temperature conditions in the future.

## 3 Conclusion

In this paper, DN ionic LBIT hydrogel has been proposed and investigated for two types of micro‐pyramid‐patterned TENG‐based hydrogel sensors, that is, the DE‐THS and the SE‐THS. The micro‐pyramid‐patterned hydrogel is utilized as both the friction layer and the conduction electrode, contributing to the high transparency (≈85% in the visible range), excellent flexibility, and wide environment tolerance (−20 to 60 °C) of the DE‐THS and the SE‐THS. Owing to the ultra‐thin thickness of the whole devices (less than 1 mm), the DE‐THS and the SE‐THS can act as self‐powered tactile sensors and achieve the sensitivities of up to 2 mV Pa^−1^ and 45.97 mV Pa^−1^, respectively, demonstrating great potential in wearable electronics and intelligent sensors. With a simple hand pressing excitation by nitrile butadiene, the SE‐THS can realize a remarkable peak power density of 20 µW cm^−2^. The transparent and flexible micro‐pyramid‐patterned hydrogel sensors have further been attached to the joint and integrated into a mask to detect human physiological motions, providing efficient and convenient methods for healthcare monitoring. The sensor shows good performance and stability in some extreme environments, including extremely low temperatures (−78.5 °C), high humidity (90%) and high‐level vacuum (−0.1 MPa). By further combining THS with a signal acquisition/process circuit, the sensor demonstrates its ability as a flexible switch button to control the electric appliance and robotic hand movements by monitoring human fingers’ gestures. The research outcomes successfully demonstrate the versatility and viability of the DN ionic LBIT hydrogel and the DE‐THS/SE‐THS for real‐world self‐powered wearable and interactive human–machine interface applications.

## 4 Experimental Section

### Materials and Characterization

Carrageenan, acrylamide, *N*′‐Methylenebis (acrylamide) (MBA), ammonium persulfate (AP), KCl, and acrylamide (AM) were obtained from Aladdin. The 99% lithium bromide was purchased from Sigma‐Aldrich. Polydimethylsiloxane (PDMS Sylgard 184) was bought from Dow Corning Inc. The 0.05 mm Ag wire was purchased from QingHe Silver Jewelry Corporation, and applied as the conductive fabric. Double sticky tapes used in this research were purchased from 3M Corporation. All reagents in this work were used in as‐received conditions without any refinement.

The transmittance of the hydrogel and DE‐THS/SE‐THS was tested using a UV–vis spectrometer (Shimadzu UV‐1780). The DSC spectra of pristine hydrogel and LBIT hydrogel were measured by using a PerkinElmer DSC 8500, with the cooling process from 20 to −115 °C at a rate of 10 °C min^−1^. XPS spectra of pristine hydrogel and LBIT hydrogel were obtained using Thermo Escalab 250Xi XPS, Thermo Fisher Scientific Inc. Micro‐structures of the samples were observed using a scanning electron microscope (SEM, VEGA 3 LMU). Because the LiBr existed in the LBIT hydrogels, they could not be freeze‐dried completely. Thus, the pristine hydrogel was chosen to coat the gold for the SEM images. CLSM image of the micro‐patterned hydrogel was acquired using the OLS 5100 Olympus. The mechanical tensile tests were performed on IBTC‐300SL tensile tester, Care Tianjin Corporation at a constant speed of 100 mm min^−1^. The Futek wiring code (WC1) was applied to measure the external force of excitation, and a mechanical shaker triggered the periodic stimulations on the DE‐THS and SE‐THS. The resistance variations of the hydrogels were measured with a digital multimeter (Keithley DMM6500). To record the output signal of the self‐powered sensor, the high‐impedance data acquisition system (NI USB‐6289 DAQ Card) was utilized in the testing platform. The surface potential of PDMS was detected by the surface potential scanner (Trek Model 347 USA). The transferred charge was measured using an electrometer system (Keithley 6514).

### Preparation of Micro‐Pyramid Patterned PDMS Films

The pre‐polymer and curing agents of PDMS were mixed at the ratio of 10:1 to prepare the PDMS solution. Then the pre‐cured solution was vacuum treated (−0.06 MPa for 30 min) to remove the air bubble inside. The uncured PDMS precursor was spin‐coated (800 rpm min^−1^ for the 30 s) on a micro‐pyramidal patterned silicon wafer to realize the thickness around 200 µm and cured on the oven at 70 °C for 2 h to form the micro‐pyramidal patterned PDMS.

### Preparation of Micro‐Pyramidal Patterned LBIT Hydrogel Film

A one‐pot polymerization method was used to synthesize the hydrogels. In the first phase, 41.0 g of deionized water with 0.005 g of MBA, 7.5 g of AM, 0.09 g of KCl, and 1.5 g of carrageenan were added into a container and magnetically stirred under the oil bath at 95 °C for 5 h. After that, the whole solution was cooled down to 75 °C and 0.0375 g of AP was subsequently added inside. The solution was stirred for several minutes and poured on the micro‐inverted pyramidal etched silicon wafer, followed by the spin coating method. Hydrogel film on the wafer was then kept at 6 °C for 1 h to format the carrageenan and heated to 95 °C for another hour to cross‐link the PAM. In the last step, the hydrogel film was immersed in the 50 wt% LiBr solution for 2 h to obtain the LBIT hydrogel with the micro‐pyramidal patterns.

### Preparation of the SE‐THS

The as‐prepared micro‐pyramidal patterned PDMS films on the silicon wafer were utilized on the bottom PDMS substrate. After adding AP into the hydrogel precursor solution and kept stirring at 75 °C for 1 min, the mixed hydrogel solution was poured on the PDMS and spin‐coated to form a thin film. The as‐prepared hydrogel film on the PDMS layer was kept at 6 °C for 1 h and heated to 95 °C for another hour for solidification. After the LBIT formation, the as‐formed hydrogel was cut into the square electrode, and a silver wire electrode was attached to the surface of this film. Another thin film of PDMS was spin‐coated and cured on the hydrogel to further encapsulate it to form the whole SE‐THS.

### Preparation of the DE‐THS

For fabrication of the DE‐THS, the micro‐pyramidal patterned hydrogel was adhered to the SE‐THS using a double sticky tape to ensure the micro‐patterned surfaces of hydrogel and PDMS were located at the opposite position. The DE‐THS was then fabricated by connecting the electrical tester between the two sliver wires in the top and bottom hydrogel electrodes.

### Study Participation

Prior to participation in the experiments, informed consent was obtained from the volunteer in all experiments.

## Conflict of Interest

The authors declare no conflict of interest.

## Supporting information

Supporting Information

Supplemental Video 1

Supplemental Video 2

Supplemental Video 3

## Data Availability

The data that support the findings of this study are available from the corresponding author upon reasonable request.
