# Supplementary material for: Ultra‐Sensitive, Deformable, and Transparent Triboelectric Tactile Sensor Based on Micro‐Pyramid Patterned Ionic Hydrogel for Interactive Human–Machine Interfaces
Source: Adv Sci (Weinh). 2022 Jan 31;9(10):2104168. doi: 10.1002/advs.202104168 (PMC8981453; doi:10.1002/advs.202104168)
Supplement: Supplementary file 1 — Supporting Information [file ADVS-9-2104168-s002.pdf]

ADVANCED SCIENCE
Open Access

Supporting Information

for *Adv. Sci.*, DOI 10.1002/advs.202104168

Ultra-Sensitive, Deformable, and Transparent Triboelectric Tactile Sensor Based on Micro-Pyramid Patterned Ionic Hydrogel for Interactive Human–Machine Interfaces

Kai Tao, Zhensheng Chen, Jiahao Yu, Haozhe Zeng, Jin Wu*, Zixuan Wu, Qingyan Jia, Peng Li*, Yongqing Fu, Honglong Chang and Weizheng Yuan**

WILEY-VCH

ADVANCED SCIENCE
Open Access

Supporting Information

for *Adv. Sci.*, DOI: 10.1002/advs.202104168

Ultra-Sensitive, Deformable and Transparent Triboelectric Tactile Sensor based on Micro-Pyramid Patterned Ionic Hydrogel for Interactive Human-Machine Interfaces

Kai Tao, Zhensheng Chen, Jiahao Yu, Haozhe Zeng, Jin Wu, Zixuan Wu, Qingyan Jia, Peng Li*, Yongqing Fu, Honglong Chang, Weizheng Yuan**

Supporting Information

Ultra-Sensitive, Deformable and Transparent Triboelectric Tactile Sensor based on Micro-Pyramid Patterned Ionic Hydrogel for Interactive Human-Machine Interfaces

Kai Tao[1], Zhensheng Chen[1], Jiahao Yu[1], Haozhe Zeng[1], Jin Wu[2], Zixuan Wu[2], Qingyan Jia[3], Peng Li[3*], Yongqing Fu[4], Honglong Chang[1], Weizheng Yuan[1*]*

Prof. K. Tao, Ma. Z. Chen, Ma. J. Yu, Be. H. Zeng, Prof. H Chang, Prof. W Yuan
Ministry of Education Key Laboratory of Micro and Nano Systems for Aerospace
Northwestern Polytechnical University
Xi’an 710072, PR China
E-mail: taokai@nwpu.edu.cn; yuanwz@nwpu.edu.cn
Prof. J. Wu, Dr. Z. Wu
State Key Laboratory of Optoelectronic Materials and Technologies and the Guangdong Province Key Laboratory of Display Material and Technology, School of Electronics and Information Technology
Sun Yat-sen University
Guangzhou 510275, PR China
E-mail: wujin8@mail.sysu.edu.cn
Prof. Q. Jia, Prof. P. Li
Frontiers Science Center for Flexible Electronics (FSCFE), Xi'an Institute of Flexible Electronics (IFE) & Xi'an Institute of Biomedical Materials and Engineering (IBME)
Northwestern Polytechnical University
Xi’an 710072, PR China
E-mail: iampli@nwpu.edu.cn
Prof. Y. Fu
Faculty of Engineering and Environment
Northumbria University
Newcastle upon Tyne, NE1 8ST, UK

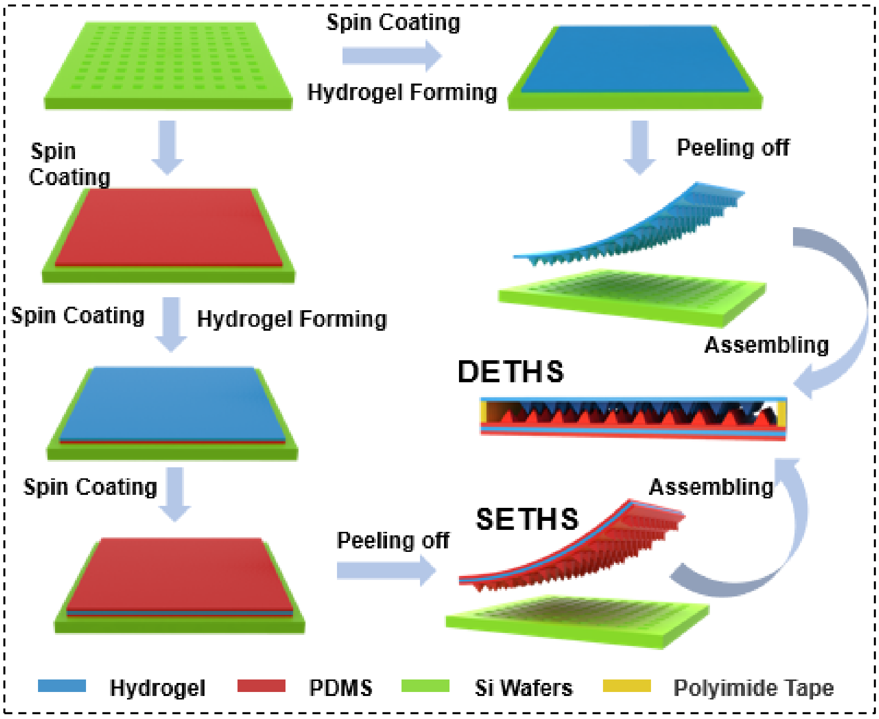

Figure S1. The fabrication process of the SE-THS and the DE-THS.

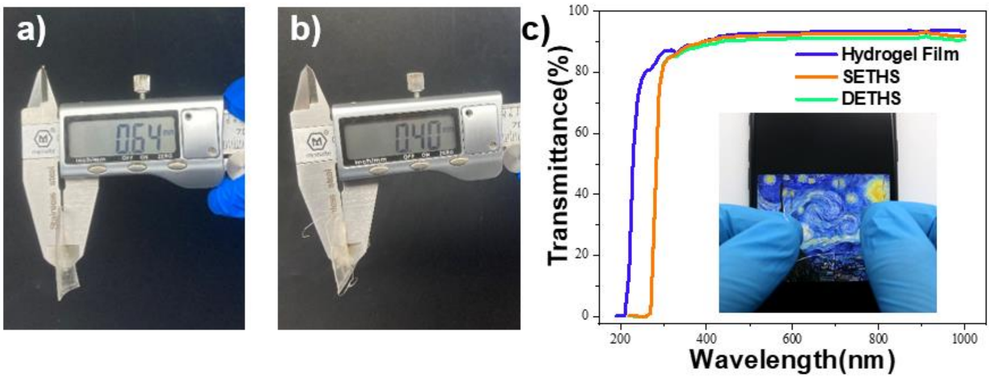

Figure S2. (a) Thickness measurement of the DE-THS. **(b)** Thickness measurement of the SE-THS. **(c)** Transmittances of the SE-THS and the DE-THS hydrogel films.

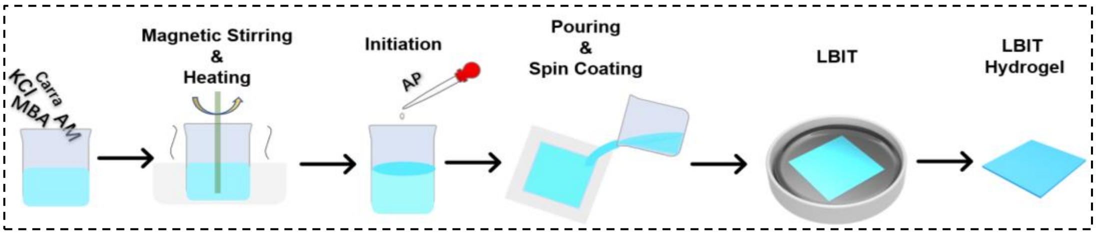

Figure S3. Synthesized method of the LBIT hydrogel.

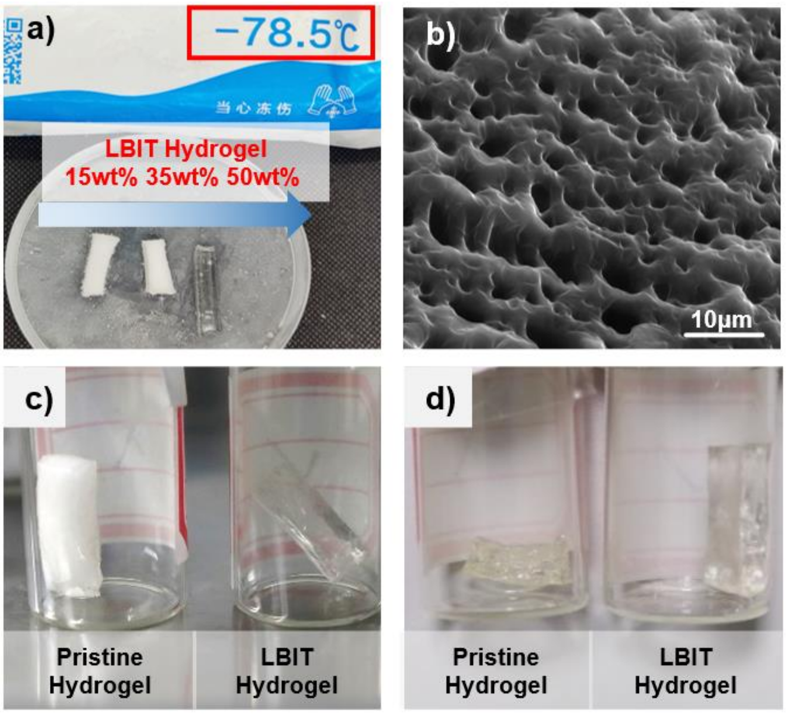

Figure S4. (a) The shape evolutions of LBIT hydrogel prototypes with different LiBr percolation percentages of 15 wt%, 35 wt% and 50 wt%. **(b)** SEM image of the freeze-dried hydrogel surface. **(c-d)** Photographs of the pristine hydrogel and LBIT hydrogel in the vacuum-freeze experiment for one minute and one month, respectively.

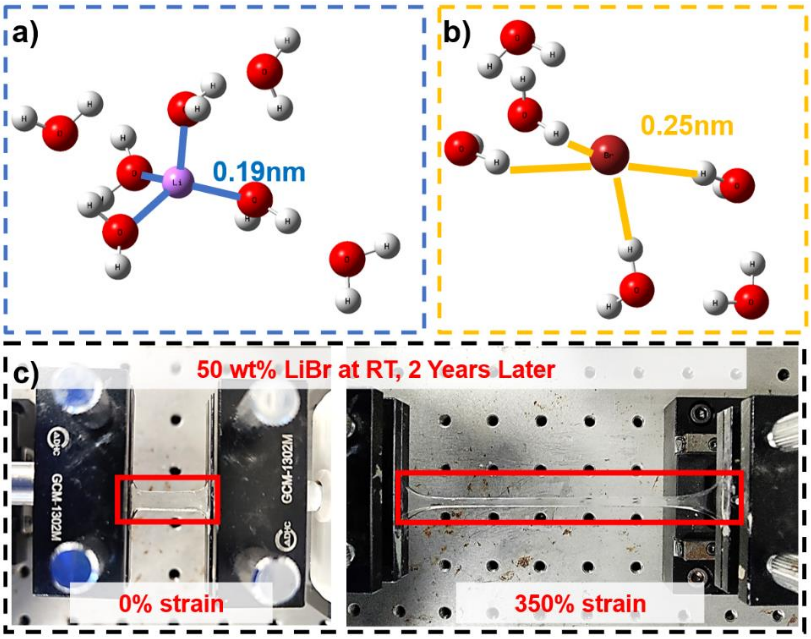

Figure S5. (a-b) Optimized structure of Li^{+}-H_2O clusters and Br^{-}-H_2O clusters, respectively. **(c)** The stretchability of the LBIT hydrogel after being stored for 2 years.

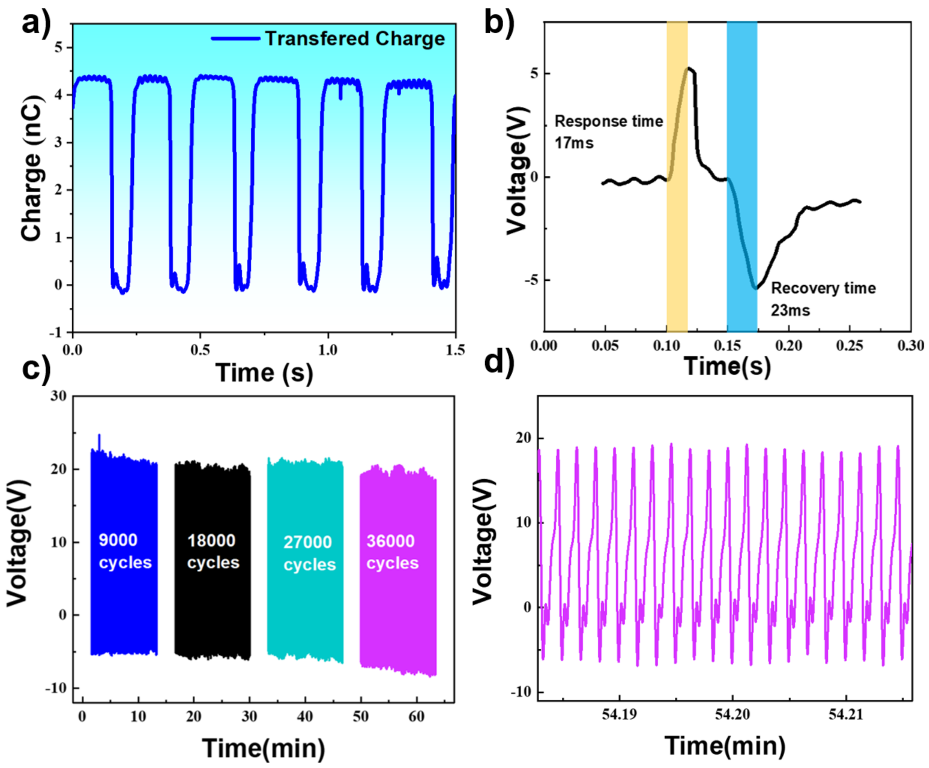

Figure S6. (a) The transferred charges of the SE-THS by the simple tapping stimulation. **(b)** The response and recovery time of the SE-THS during the finger-tapping process. **(c)** Output performance stability of the SE-THS after continuously operating for 36000 cycles. **(d)** Enlarged view of the long-term output waveforms.

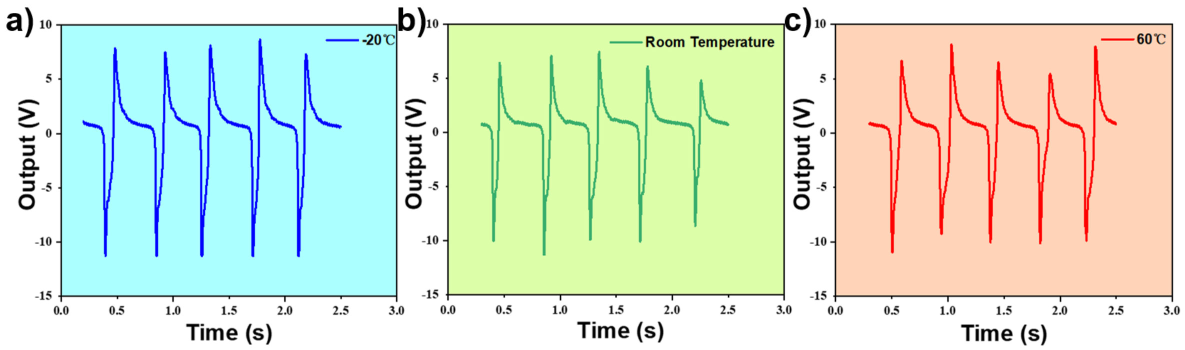

Figure S7. Output performance of the DE-THS at: **(a)** −20 ℃; **(b)** Room temperature; **(c)** 60 ℃.

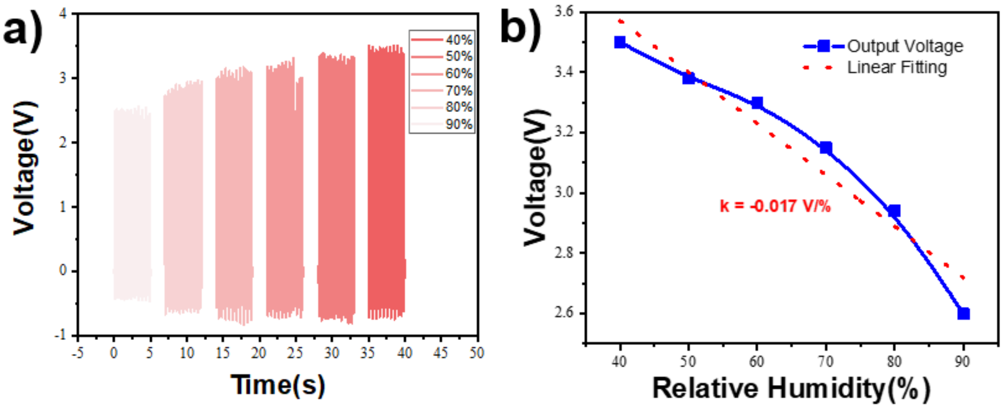

Figure S8. (a) Voltage variations of the DE-THS at different humidity levels. **(b)** The relationship between the relative humidity and the output voltages of the DE-THS.

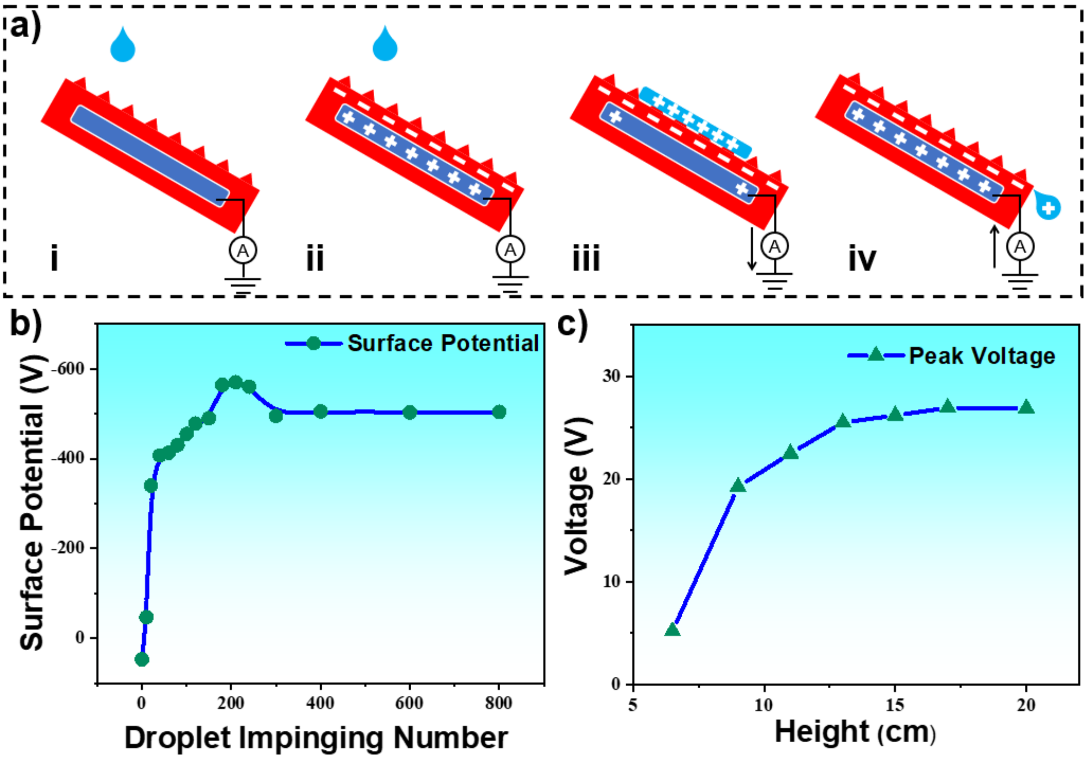

Figure S9. The waterdrop detection experiments by the LBIT hydrogel sensor (SE-THS): **(a)** the self-power generation principle during the water-PDMS contact electrification process. **(b)** Relationship between the surface potential and the impinging droplet numbers. **(c)** The dependence of the maximum output voltage on the height of the droplet.

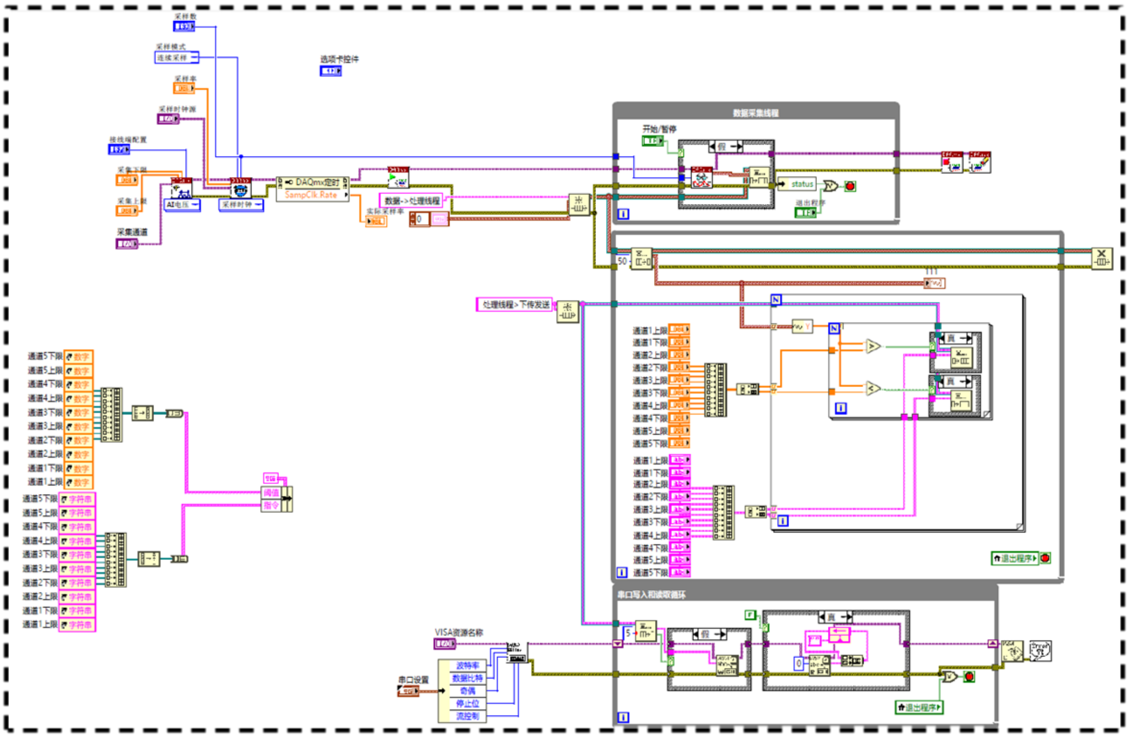

Figure S10. The logical diagram of the customized signal acquiring/processing procedure in the LabView Software.

Table S1 XPS elemental analysis of DN ionic hydrogel

Sample	C	O	N	S	K	Cl	Li	Br
Pristine Hydrogel	62.57	20.17	16.06	0.76	0.3	0.14	0	0
LBIT Hydrogel	56.48	19.44	8.99	0.6	0.02	0.06	8.98	5.43

Table S2 Comparisons of the-state-of-art thin-film flexible sensors

Materials	Operation Principle	Transparency	Thickness	Self-Power	Temperature Tolerance	Pressure Sensitivity
PEFE/Graphene/PDMS/Ag NWs[1]	Piezo-resistance	No	5 mm	Yes	Yes	0.015/Pa
VHB/TPU/PDMS/Ag NWs[2]	Triboelectric	48%	89μm	Yes	No	9.973mV/Pa
VHB/PAM Hydrogel[3]	Triboelectric	~90%	2.2mm	Yes	Yes	0.013mV/Pa
Patterned PDMS/ PAMPS Ion gel[4]	Triboelectric	83%	0.2mm	Yes	No	0.528mV/Pa*
RGD/Ti_2C-MXene/Cu[5]	Piezoresistive	No	-	No	No	0.57/Pa
Porous PDPU/Ag BM[6]	Triboelectric	No	4mm	Yes	No	17mV/Pa
Latex/PUA sliver flakes liquid metal[7]	Triboelectric	No	300μm	Yes	No	-
Cu/PDMS/P (VDF-TrFE)[8]	Triboelectric	No	0.59 mm	Yes	Yes	0.0567mV/Pa
Au/PDMS/Ag NWs/Al[9]	Triboelectric	No	-	Yes	No	0.0003/Pa
ITO/PET/PDMS[10]	Capacitive	-	-	No	No	0.0006/Pa
Cellulose hydrogel/VHB[11]	Triboelectric	94%	1.5 mm*	Yes	Yes	0.269 mV/Pa*
Poly (acrylic acid) hydrogel[12]	Piezoresistive	No	1.5 mm	No	No	0.0005/Pa*
PDMS/ITO/PET[13]	Capacitive	-	-	No	No	0.000055/Pa
This work	Triboelectric	~90%	400μm	Yes	Yes	45.97mV/Pa

* is estimated from the article.- is not mentioned.

Section S1: Theoretic calculated result about the output promotion from DE-THS (plain PDMS and pyramidal patterned hydrogel).

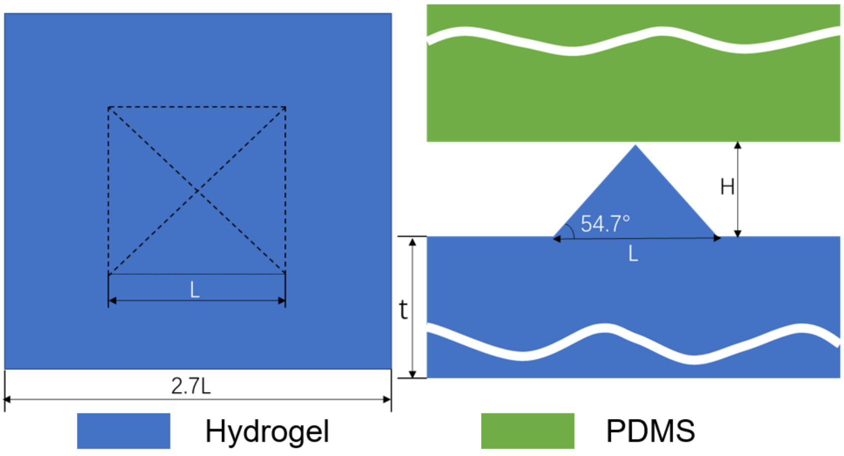

Figure S11. Geometric parameters of pyramidal PDMS at initial state.

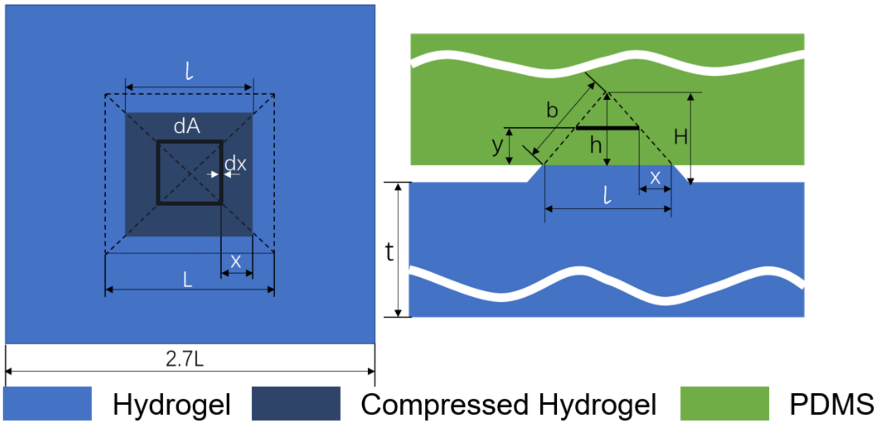

Figure S12. Variables of pyramidal hydrogel at pressed state.

Due to the strength of PDMS is much higher than that of the hydrogel, we neglect the change of PDMS surface area when pressed.

The pyramids were arranged in an array, so one unit block was studied firstly. As shown in Figure S11, L means the base length of pyramids and 2.7L is the side length of one unit which is calculated by the base length and the gap between pyramids. H stands for the height of a pyramid. t is the thickness of the hydrogel. Owing to the inherent characteristics of

anisotropic etching silicon by using TMAH, the side angle of a pyramid is usually 54.7°. (Parameters utilized in this research: $E_{hydrogel}$=300 kPa, t=200 μm, L=25 μm, $\varepsilon=2.6\times8.85\times10^{-12}$ F m^{-1}, σ=2.2 μC m^{-2})

When the external force is exerted (as is demonstrated in Figure S12), the repulsive force makes equilibrium with the external force. Hydrogel and PDMS contact each other and the pyramids on hydrogel could be compressed. At a compressed depth of **h**, the surface area of contact region:

$$A_{contact} = 4\times\frac{l\times b}{2} = 4\times\frac{h}{\tan(54.7°)}\times\frac{h}{\sin(54.7°)} = 3.47h^2 \quad (1)$$

Because the compressed depth varies from the distance from center, we differentiate the contact part of the pyramid according to the distance **x** from the edge and calculate the repulsive force. Where **dA** represents the area of the micro-element, **dx** is its thickness, and **E** is the Young's modulus of hydrogel.

The repulsive force of the target square:

$$d_F = \left[\frac{E}{t}\times 4\times(l-2x)\times dx\right]\times y = 5.65\left(\frac{E}{t}\right)(lx-2x^2)\,dx \quad (2)$$

The repulsive force at the compressed depth of **h** could be studied by integrating **x** from 0 to 0.5l:

$$\int_0^F dF = 5.65\times\left(\frac{E}{t}\right)\times\int_0^{0.5l}(lx-2x^2)dx \quad (3)$$

$$F = 5.56\left(\frac{E}{t}\right)\left(\frac{1}{2}lx^2-\frac{2}{3}x^3\right)_0^{0.5l} = 0.668\left(\frac{E}{t}\right)h^3 \quad (4)$$

The repulsive force **F** has the following relationship with the external pressure:

$$F = p * A_{unit} = p \times \left(2.7 L^2 \right) \tag{5}$$

Where **A_{unit}** stands for the area of a square unit.

Therefore, we could obtain the dependence of **h** on external pressure **p**:

$$h = 2.23 \left(\frac{tpL^2}{E} \right)^{\frac{1}{3}} \tag{6}$$

The area of contact region has the following relationship with p:

$$A_{contact} = 3.47 h^2 = 17.21 \left(\frac{tpL^2}{E} \right)^{\frac{2}{3}} \tag{7}$$

The above discussion is focused on one unit, the total contact area of the whole device:

$$\begin{aligned} A_{device\ contact} &= n \times A_{connect} = \frac{A_{device\ contact}}{A_{unit}} \times A_{connect} = \frac{A_{device\ contact}}{7.29 L^2} \times 17.21 \left(\frac{tpL^2}{E} \right)^{\frac{2}{3}} \\ &= 2.36 A_{device\ contact} \left(\frac{tp}{EL} \right)^{\frac{2}{3}} \end{aligned} \tag{8}$$

The total amount of transferred charges of the whole device:

$$Q = \sigma \times A_{device\ contact} = 2.36 \sigma A_{device\ tatal} \left(\frac{tp}{EL} \right)^{\frac{2}{3}} \tag{9}$$

Open circuit voltage (**Voc**) is determined by the transferred charges **Q** and capacitance **C**:

$$V_{OC} = \frac{Q}{C} = \frac{2.36 \sigma A_{device\ total} \left(\frac{tp}{EL} \right)^{\frac{2}{3}}}{\varepsilon A_{device\ total} / t} = 2.36 \left(\frac{\sigma t}{\varepsilon} \right) \left(\frac{tp}{EL} \right)^{\frac{2}{3}} \tag{10}$$

Video S1 THS plays the role of button switch in two modes.

Video S2 Robotic hand control with five THSs.

Video S3 Gestures control of the robotic with five THSs.

References

[1] Y. Wang, H. Wu, L. Xu, H. Zhang, Y. YangZ. L. Wang. Hierarchically patterned self-powered sensors for multifunctional tactile sensing. *Sci Adv*, **2020,** *6*, eabb9083.

[2] Y. Jiang, K. Dong, X. Li, J. An, D. Wu, X. Peng, J. Yi, C. Ning, R. Cheng, P. YuZ. L. Wang. Stretchable, Washable, and Ultrathin Triboelectric Nanogenerators as Skin‐Like Highly Sensitive Self‐Powered Haptic Sensors. *Adv. Funct. Mater.*, **2020,** *31*, 2005584.

[3] X. Pu, M. Liu, X. Chen, J. Sun, C. Du, Y. Zhang, J. Zhai, W. HuZ. L. Wang. Ultrastretchable, transparent triboelectric nanogenerator as electronic skin for biomechanical energy harvesting and tactile sensing. *Sci Adv*, **2017,** *3*, e1700015.

[4] G. Zhao, Y. Zhang, N. Shi, Z. Liu, X. Zhang, M. Wu, C. Pan, H. Liu, L. LiZ. L. Wang. Transparent and stretchable triboelectric nanogenerator for self-powered tactile sensing. *Nano Energy*, **2019,** *59*, 302.

[5] X.-F. Zhao, C.-Z. Hang, H.-L. Lu, K. Xu, H. Zhang, F. Yang, R.-G. Ma, J.-C. WangD. W. Zhang. A skin-like sensor for intelligent Braille recognition. *Nano Energy*, **2020,** *68*, 104346.

[6] J. Xiong, G. Thangavel, J. Wang, X. ZhouP. S. Lee. Self-healable sticky porous elastomer for gas-solid interacted power generation. *Sci Adv*, **2020,** *6*, eabb4246.

[7] K. Parida, G. Thangavel, G. Cai, X. Zhou, S. Park, J. XiongP. S. Lee. Extremely stretchable and self-healing conductor based on thermoplastic elastomer for all-three-dimensional printed triboelectric nanogenerator. *Nat Commun*, **2019,** *10*, 2158.

[8] J. Yu, X. Hou, J. He, M. Cui, C. Wang, W. Geng, J. Mu, B. HanX. Chou. Ultra-flexible and high-sensitive triboelectric nanogenerator as electronic skin for self-powered human physiological signal monitoring. *Nano Energy*, **2020,** *69*, 104437.

[9] L. Lin, Y. Xie, S. Wang, W. Wu, S. Niu, X. WenZ. L. Wang. Triboelectric active sensor array for self-powered static and dynamic pressure detection and tactile imaging. *ACS Nano*, **2013,** *7*, 8266.

[10] S. C. Mannsfeld, B. C. Tee, R. M. Stoltenberg, C. V. Chen, S. Barman, B. V. Muir, A. N. Sokolov, C. ReeseZ. Bao. Highly sensitive flexible pressure sensors with microstructured rubber dielectric layers. *Nat Mater*, **2010,** *9*, 859.

[11] Y. Hu, M. Zhang, C. Qin, X. Qian, L. Zhang, J. ZhouA. Lu. Transparent, conductive cellulose hydrogel for flexible sensor and triboelectric nanogenerator at subzero temperature. *Carbohydr. Polym.*, **2021,** *265*, 118078.

[12] X. Shen, L. Zheng, R. Tang, K. Nie, Z. Wang, C. JinQ. Sun. Double-Network Hierarchical-Porous Piezoresistive Nanocomposite Hydrogel Sensors Based on Compressive Cellulosic Hydrogels Deposited with Silver Nanoparticles. *ACS Sustainable Chemistry & Engineering*, **2020,** *8*, 7480.

[13] C. Mahata, H. Algadi, J. Lee, S. KimT. Lee. Biomimetic-inspired micro-nano hierarchical structures for capacitive pressure sensor applications. *Measurement*, **2020,** *151*, 107095.
